# 264. Resident Macrophages of the Amnion: Characterization and Response to Ascending Bacterial Pathogens

**DOI:** 10.1093/ofid/ofad500.336

**Published:** 2023-11-27

**Authors:** Eva Fandozzi, Caroline Smith, Christina Megli

**Affiliations:** University of Pittsburgh School of Medicine, Pittsburgh, Pennsylvania; University of Pittsburgh School of Medicine, Pittsburgh, Pennsylvania; Magee-Womens Research Institute, Pittsburgh, Pennsylvania

## Abstract

**Background:**

The primary organ of pregnancy, the placenta, exists as a maternal-fetal barrier and displays remarkable immunoactivity. This barrier is effective against multiple pathogens, yet, the underlying mechanisms of immune regulation and pathogen defense of this barrier are not well understood. The inner layer of the fetal membrane, the amnion, has been proposed to be a significant barrier to infection. The amnion has been reported to contain CD14+ cells, but the immune activity and localization of these cells are poorly characterized. As a monocyte marker, these CD14+ cells may be endemic to the amnion and involved in antimicrobial defenses.

**Methods:**

We isolate macrophages from amnion explants of fetal membranes collected at cesarean section in the absence of labor and infection using a StemSep Human CD14+ Selection Kit. These cells are characterized using flow cytometry and FACS. In addition, mCherry labeled *L. monocytogenes* (LM) or CFSE labeled *S. agalactiae* (GBS) were used to infect intact explants and infectivity was quantified by colony forming units. Immunofluorescence staining was used to localize CD14. Bacteria colonization with CD14 was visualized in infected tissue explants. CD86 and CD163 were used as M1 and M2 markers respectively. Imaging was performed using the STELLARIS confocal microscope.

**Results:**

CD14+ cells exist in healthy amniotic tissue explants and localize to the mesenchymal layer. Amnion CD14+ cells also co-localize with CD86 and without CD163 suggesting a distinct phenotype from other placental macrophages (Hoffbauer cells and PAMM subsets). Amnion explants have decreased growth of LM and GBS in comparison to chorion and chorionic villi. LM and GBS co-localized with CD14+ cells in the amnion.

CD14+ macrophages are present in healthy amnion explants

**Figure:**
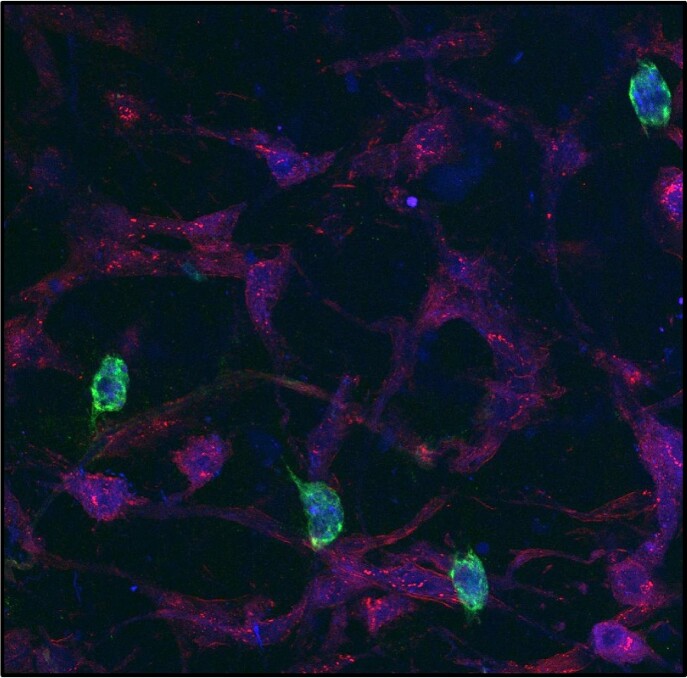

Immunofluorescence staining of healthy human amnion. CD14 (green) positive cells localize to the mesenchymal layer of the amnion. Nuclei and cytoplasmic actin are stained with DAPI (blue) and phalloidin (red) respectively.

GBS localizes to the mesenchymal layer of the amnion

**Figure:**
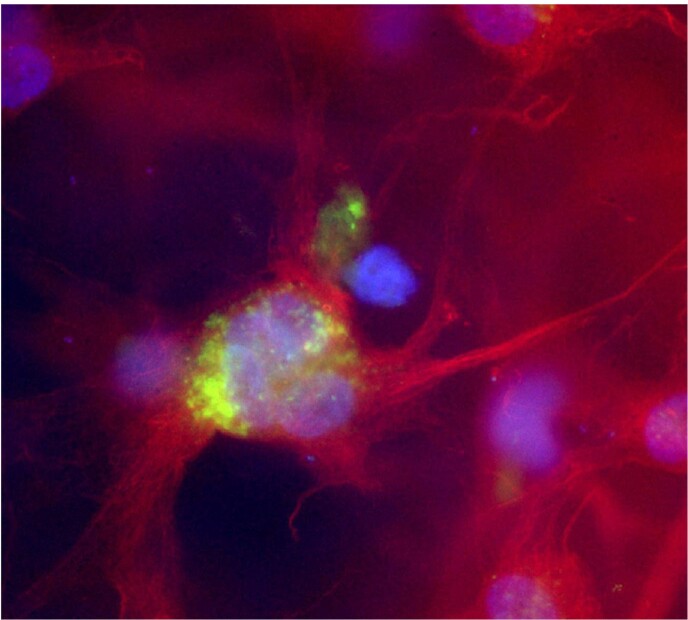

Immunofluorescence staining of healthy human amnion. GBS (green) localizes to the mesenchymal layer of the amnion. Nuclei and cytoplasmic actin are stained with DAPI (blue) and phalloidin (red) respectively.

**Conclusion:**

We present the novel finding of resident macrophages in the amnion and demonstrate the presence of amnion macrophages in the absence of infection. In addition, we demonstrate that these macrophages express CD86 and co-localize with bacteria that can be vertically transmitted during pregnancy, including LM and GBS. Further characterization of the regulation of these cells may provide insight into the immunomodulatory function of the fetal membrane.

**Disclosures:**

**All Authors**: No reported disclosures

